# Fe-functionalized paramagnetic sporopollenin from pollen grains: one-pot synthesis using ionic liquids

**DOI:** 10.1038/s41598-020-68875-6

**Published:** 2020-07-20

**Authors:** C. Chiappe, M. J. Rodriguez-Douton, M. C. Mozzati, D. Prete, A. Griesi, L. Guazzelli, M. Gemmi, S. Caporali, N. Calisi, C. S. Pomelli, F. Rossella

**Affiliations:** 1 Dipartimento di Farmacia, Università di Pisa, Via Bonanno 33, 56126 Pisa, Italy; 2 Dipartimento di Fisica, Università di Pavia, Via Bassi 6, 27100 Pavia, Italy; 3 NEST, Scuola Normale Superiore and Istituto Nanoscienze-CNR, Piazza San Silvestro 12, 56126 Pisa, Italy; 4 Center for Nanotechnology Innovation@NEST, Istituto Italiano di Tecnologia, Piazza San Silvestro, 12, 56127 Pisa, Italy; 5 Department of Chemistry, Life Sciences and Environmental Sustainability, University of Parma, Parco Area delle Scienze 17/A, 43124 Parma, Italy; 6 Dipartimento di Ingegneria Industriale, Università di Firenze, Via di S. Marta 3, 50129 Firenze, Italy; 7 INSTM, Via Giusti 9, 50123 Firenze, Italy

**Keywords:** Chemistry, Materials science, Physics

## Abstract

The preparation of Fe-decorated sporopollenins was achieved using pollen grains and an ionic liquid as solvent and functionalizing agent. The integrity of the organic capsules was ascertained through scanning electron microscopy studies. The presence of Fe in the capsule was investigated using FT-IR, X-ray photoemission spectroscopy and energy-dispersive X-ray spectroscopy. Electron paramagnetic resonance and magnetization measurements allowed us to demonstrate the paramagnetic behavior of our Fe-functionalized sporopollenin. A few potential applications of pollen-based systems functionalized with magnetic metal ions via ionic liquids are discussed.

## Introduction

Highly organized and complex 3D microstructures hold great promise for manifold applications across nanotechnologies as carriers, scavengers or catalysts, to name a few possible functional uses^1–4^. Driven by these exciting prospects, great efforts have been devoted in the last few years to the preparation of polymeric microcapsules through different routes, including template-assisted methods, self-assembly of block copolymer processes, and interfacial mini-emulsion polymerization methods^5–8^. However, the synthesis of such structures is generally costly and difficult, especially when obtaining microcapsule of uniform size distribution and large inner cavity is crucial. In this context, innovative strategies are progressively moving beyond artificial material synthesis and looking to nature for inspiration. In particular, scientists have started to appreciate the myriad of unique, widely available biological systems and their microscale and nanoscale architectures that might be useful for the design and fabrication of functional materials. For instance, viruses, large globular proteins like ferritin, chromatids are nanoscale structures; Larger—still microscale—structures are pollen grains.

In this scenario, pollen grains represent a very promising source of 3D microstructures with desirable morphologies: they can indeed be easily sourced in large amounts from plants and can provide, through cost-effective approaches, polymeric microcapsules with a wide variety of shape and size. These features, and most importantly their consistency which is guaranteed by the species specificity, are crucial and difficult to obtain with purely synthetic materials^9–11^. In pollen grains, the male partner in the reproductive process, the genetic material is protected by a double-layered wall, which consists of an inner layer (*intine*) and an outer layer (*exine*). The former is constituted mainly of cellulose, hemicellulose and pectin, whereas the latter, known as sporopollenin (SP) is composed largely of a biopolymer, whose chemical structure is still unclear, containing only carbon, hydrogen and oxygen atoms. This polymer is characterized by remarkable strength and elasticity. Furthermore, it shows an extraordinary resistance to chemical degradation or dissolution while being at the same time readily amenable to derivatization due to the presence of a network of functional groups (carboxyl, carbonyl, alcoholic and ether functions).

To exploit the 3D microstructures of SP as scaffolds or templates, the polymer must be isolated through accurate purification processes that usually consist in the use of aggressive and/or corrosive chemical agents, as well as organic solvents^12^. Recently, some of us reported a novel procedure for the separation of sporopollenin from the other pollen components, using ionic liquids (ILs)^13^. ILs are organic salts which are liquid below 100 °C and, thanks to the possibility to tailor their physico-chemical properties by changing the constituting ion, found use in a wide range of different applications during the last two decades^14–19^.

The capability to achieve polymer isolation and at the same time to control the functionalization of these complex organic systems by giving them special properties, represent the key ingredient for future applications. This approach is not necessarily limited to their use as carriers, but can be extended to other uses as well. Sporopollenin has indeed attracted great attention as an agent able to remove heavy metals from water: this is due to its excellent chemical and physical resistance and to the fact that it features many organic groups able to interact with metal ions or to be easily functionalized^20,21^. Nonetheless, their intrinsic adsorptive characteristics can be further improved by introducing suitable functional groups. Recently, a novel Fe-modified sporopollenin prepared through chemical precipitation of iron oxide magnetic particles (Fe_3_O_4_) on the sporopollenin surface has been shown. The practical application of such material was the removal of Pb^2+^ in an aqueous environment^22^.

Here, we use an environmentally friendly chemical route to a Fe-modified sporopollenin based on a hydrophobic IL, 1-butyl-3-methylimidazolium tetrachloroferrate, [bmim][FeCl_4_]. This material was used both to purify and further to functionalize sporopollenins (SP) with Fe-ions. The presence of the metal in the SP microcapsules as well as their morphology was investigated using FT-IR spectroscopy, X-ray photoemission spectroscopy (XPS) and scanning electron microscopy (SEM). The accurate magnetic study based on electron paramagnetic resonance (EPR) and superconducting quantum interference device (SQUID) measurements reveals near perfect paramagnetism in the Fe-decorated sample. Our work experimentally demonstrates that the decoration of SP with Fe-ions using ionic liquids provides a formidable—green, facile and low cost—chemical route to a novel class of organic-based paramagnetic systems at the micro-scale.

## Results and discussion

### Fe-decorated SPs: FT-IR study

**Figure 1:**
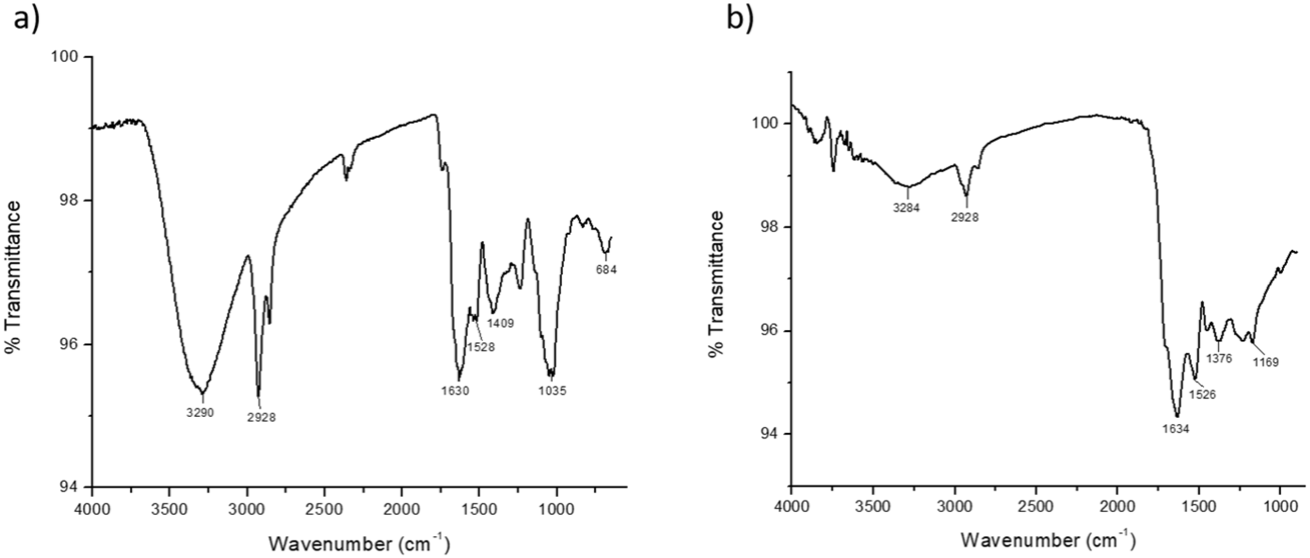
ATR FT-IR of (**a**) pristine *Populus deltoides* and (**b**) IL-treated pollen grains.

The dark-brown sporopollenin microcapsules arising from the treatment of *Populus deltoides* pollen grains with [bmim][FeCl_4_] were analyzed by ATR FT-IR. As previously observed using other ILs, the FT-IR spectrum of the recovered material is significantly modified with respect to the pristine pollen grains, with a number of peaks reduced in size or altogether missing. In particular, after IL treatment the strong absorption bands at 1,035 cm^−1^ (C–O) and 3,280 cm^−1^ (O–H) attributable to carbohydrates and cellulose (Fig. 1a) practically disappear, and the stretching modes of the aliphatic C–H bonds undergo to a drastic reduction (Fig. 1b). The weak absorption at 3,204 cm^−1^ is probably due to the presence of water. Furthermore, significant modifications can be observed also in the region between 1,750 and 1,200 cm^−1^, where usually the vibrational modes of lipids (1739 cm^-1^, C=O stretching; 1,413 cm^−1^, CH_2_ deformation; 1,236 cm^−1^, C–O stretching) and proteins (1,630 cm^−1^, amide I; 1,530 cm^−1^, amide II) are found, i.e. the *intine* components of pollen grains, and the vibrations of the sporopollenin (1,712–1,650 cm^−1^, C=O, 1,605, 1,515, 1,171, aromatic rings). IL treatment removes or reduces peaks related to lipids and proteins, hence the aromatic peaks around 1,610 cm^−1^ emerge as well as the absorption band around 1,700 cm^−1^, attributable to a conjugated C=O functional group^23,24^. Finally, it is worth to note that the absorption bands at 1634 and 1,520 cm^−1^ are still present, which could suggest an incomplete removal of the proteic component. However, the possible complexation *of the IL iron* to sporopollenin carboxyl groups (see below) could produce strong absorption bands in the same region (1,665–1,470 cm^−1^) due to ν_as_(OCO), ν_s_(OCO) and δ(OCO).

### Fe-decorated SPs: chemical and morphological analysis

**Figure 2:**
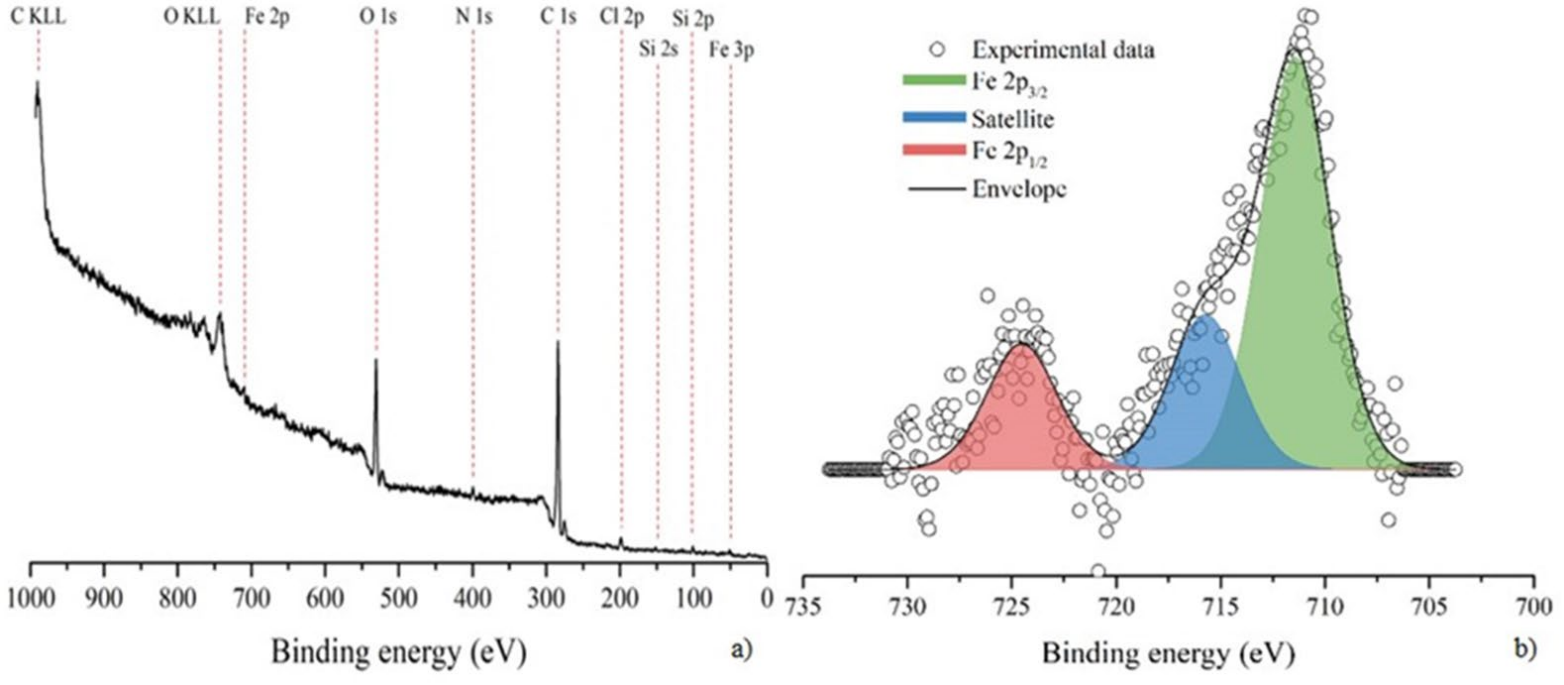
(**a**) wide, low-resolution spectrum and (**b**) high-resolution spectrum of Fe 2*p* core transition collected on the IL-treated pollen grains.

Wide scan overview and high-resolution XPS spectra of characteristic Fe 2*p* core transition of the IL-treated pollen grains, are depicted in Fig. 2a and b, respectively. The surface of the IL-treated pollen grains, as determined by XPS, is dominated by the elements constituting the organic capsule, mainly carbon (1*s* transition at 284.8 eV) and oxygen (1*s* transition at 531 eV) (see Fig. 2a)^25^. The smaller peaks observable in the spectrum were attributed to iron (2*p*3/2 transition at 710 eV and 3*p* transition at 53 eV), nitrogen (1*s* transition at 400 eV) and chlorine (2*p*3/2 transition at 198 eV)^26^. Silicon was also recognized (2*s* transition at 152 eV and 2*p* transition at 101 eV) probably due to silicon trace used to grease flask’s neck. High-resolution spectra of iron 2*p* doublet are depicted in Fig. 2b. The spectra is composed by the Fe 2*p* doublet (2*p*3/2 and 2*p*1/2), spin–orbit splitting 13.1 eV^27^ and a satellite peak (blue component in Fig. 2b)^28^ which is characteristic of the presence of high spin iron species. The energy of the 2*p*3/2 core transition of iron resulted 711.3 eV and it is reasonably attributed to pristine Fe^3+^ chloride presents in the IL^29^.

**Figure 3:**
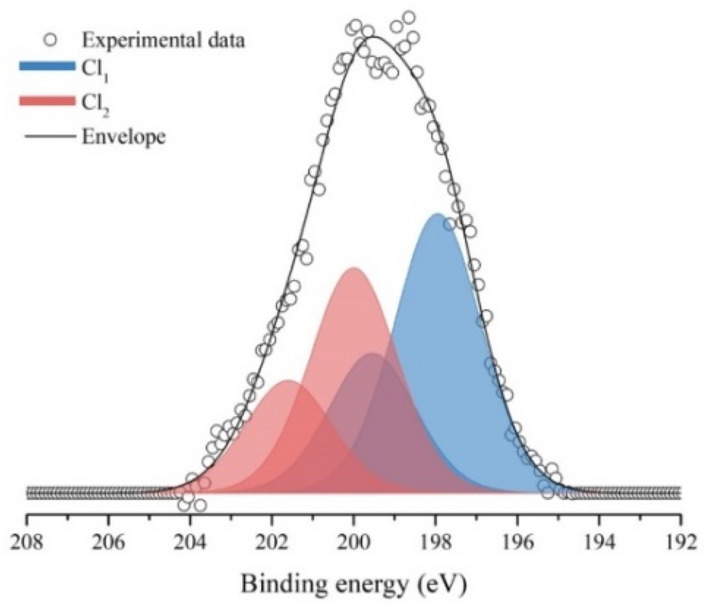
XPS peak relative to chlorine 2*p* core transition.

It is worth to note that also chlorine is detected at the surface of the IL-treated pollen grains and its high-resolution spectrum of the 2*p* core transition is reported in Fig. 3. In this case the peak requires two components to be fitted (named Cl_1_ at 198.0 eV and Cl_2_ at 200.0). At this moment, these components cannot be unambiguously attributed.

In Table 1 the experimental and theoretical relative amount of iron and chlorine are reported. The experimental amount of each element was obtained by applying literature atomic sensitivity factors^26^ to the area of XPS peaks. The only source for these elements is the IL. In the pure IL the ratio between Fe and Cl is 1:4 but in the surface of IL-treated pollen grains this ratio decreases to 1:2. That can be reasonably attributed to the partial substitution of chlorine by some other groups (mainly oxyls and hydroxyls) present in the pollen.

**Table 1:** Surface elemental composition as determined by XPS and expected theoretical amount of iron and chlorine.

Component	Position (eV)	Experimental relative amount (%)	Theoretical relative amount (IL) (%)
Fe	711.3	34	20
Cl	198.0 and 200.0	66	80

**Figure 4:**
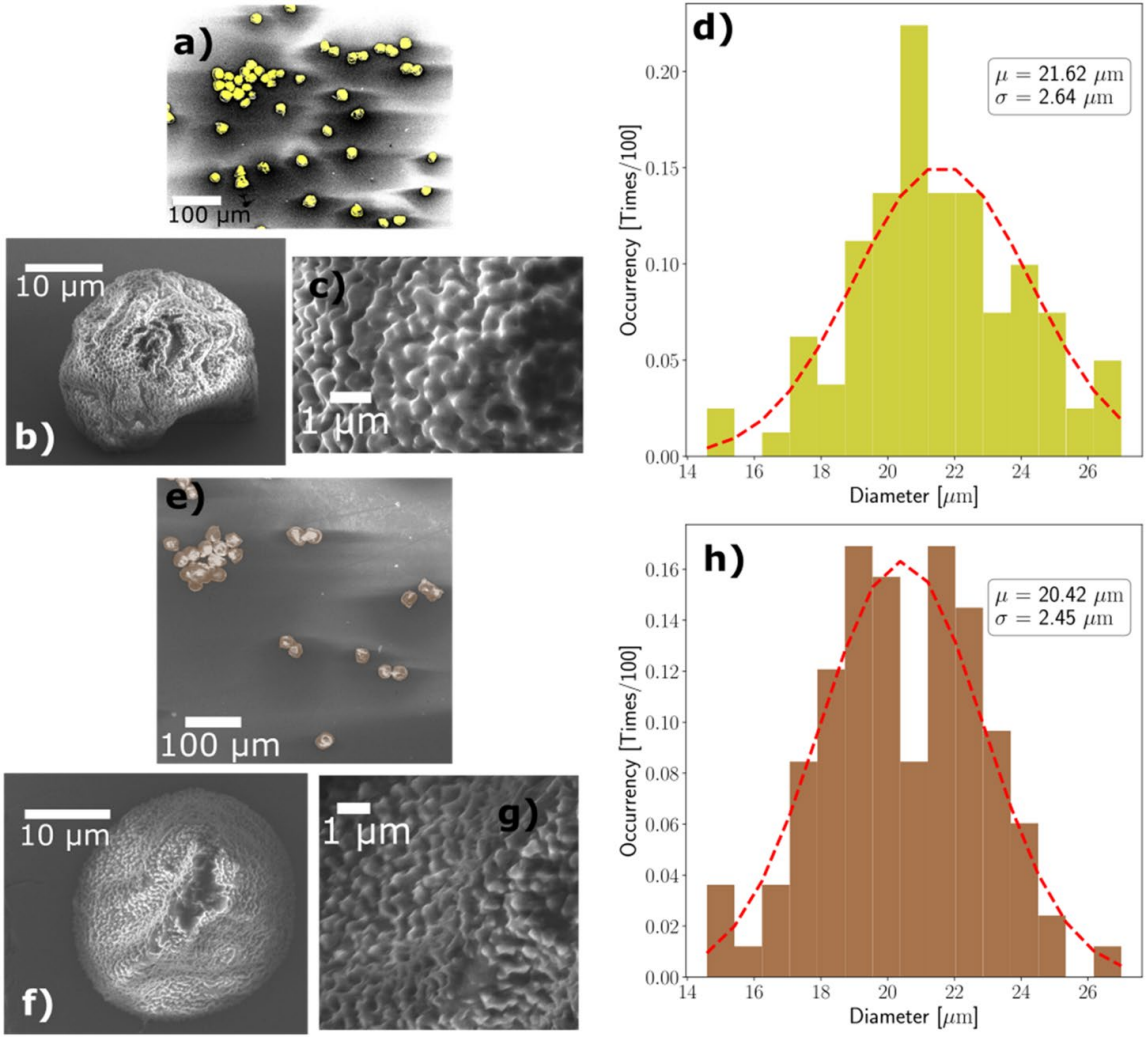
Comparison between pristine pollen grains and Fe-decorated sporopollenins. (**a**–**c**) report scanning electron micrographs of pristine pollen grains at different length scales: particles are highlighted in yellow in the false-color micrograph in panel (**a**), while panel (**b**, **c**) allow to appreciate the porous surface of non-treated pollen grains. Panel (**d**) reports the diameter distribution of the particles under observation (100 grains were considered in order to retrieve the reported distribution) along with a gaussian fit resulting in $\mu =21.62 \upmu m$ and $\sigma =2.64 \upmu m$ values for the mean and standard deviation respectively. (**e**–**h**) report the corresponding scanning electron micrograph and statistical analysis for Fe-decorated sporopollenins. In this latter case, the gaussian fit (obtained taking into account measurements of the diameter for 100 treated particles) returns values $\mu =20.42 \upmu m$ and $\sigma =2.45 \upmu m$ for the mean and standard deviation of the distribution of diameters. A direct comparison between the reported panels allow to appreciate that no morphological nor dimension-wise changes are induced by the Fe decoration of sporopollenins from non-treated pristine pollen grains.

The shape and morphology of Fe-decorated individual pollen grains have been addressed using scanning electron microscopy (SEM). In Fig. 4a–c and e–g we report SEM micrographs at increasing magnification from a pristine and Fe-functionalized sporopollen grain, respectively. The images allow to appreciate the morphological features of both samples down to the sub-micron scale: the latter is characteristic of the corrugations and of the roughness displayed by the surface of the pollen grain. Noticeably, the Fe-functionalization preserves the overall spherical 3D structure typical of individual grains of the pristine (not functionalized) pollen, as well as their morphology and dimensions. The unchanged morphological features are particularly evident from the very high magnification images (Fig. 4c, g), displaying the sawtooth-like nanotexturing already reported for pristine pollen grains^13^. Here it is worth noting that, in general, the accurate SEM investigation of organic samples presents various challenges and limitations. In fact, the high-energy electrons from the scanned beam may undergo unwanted interactions with atoms and electrons at the surface of the organic material, and may also be trapped and accumulated locally on the surface. This electrical charging of the sample eventually induces spurious effects in the generation of the secondary electrons whose detection is at the base of the image generation. Overall, this process can detrimentally affect the lower threshold for spatial resolution, which will exceed several tens of nanometers or even more. Moreover, the microscopic events leading to image distortion occur on their own space and time scale so that the image can on occasion get dynamically worse until basically a black background containing no information is returned. While we had to face these issues during SEM investigation of pristine pollens, we experienced instead a very good focusing of the electron beam during the entire duration of each session of imaging: no charging and no dynamical distortion of the image, even exploring a wide range of applied voltage in the operation of the SEM (5 kV to 20 kV). We tentatively ascribe this experimental evidence to the incorporation of iron in the SP that very likely improves the electrical transport properties of the pollen grain and eventually promotes the isotropic redistribution of the electrical charge on the surface of the grain. For what regards any possible changes involving the dimensions of grains upon Fe functionalization, in Fig. 4d, h statistical studies of diameter distributions for pristine grains as well as Fe-decorated sporopollenins are reported. The histogram in Fig. 4d (yellow color) reports the diameter distribution for 100 pristine pollen grains and the red dashed line represents a gaussian fit performed on these data. The best fit resulted in a distribution having $\mu =21.62 \upmu m$ as the mean value and $\sigma =2.64 \upmu m$ as standard deviation. Similarly, Fig. 4h reports corresponding data for Fe-decorated sporopollenins. In this case, the resulting values for the mean value and standard deviation of normal distribution describing the measured diameter values are $\mu =20.42 \upmu m$ and $\sigma =2.45 \upmu m$, meaning that there is no statistically relevant deviations between the diameter distribution of pristine pollen grains and Fe-decorated sporopollenins.

**Figure 5:**
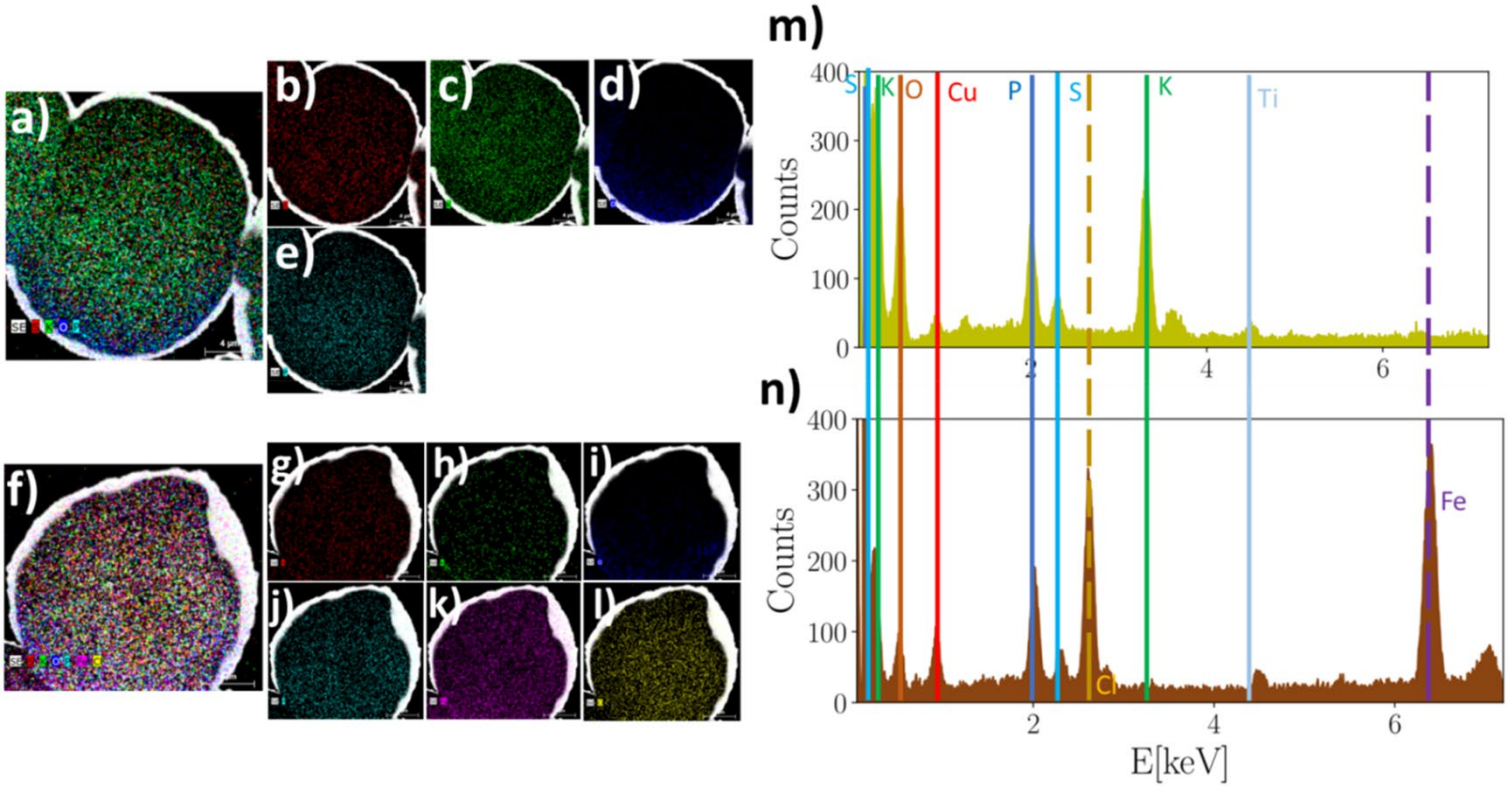
Material composition analysis of pristine pollen grains and Fe-decorated sporopollenins measured by means of EDX technique. Panel (**a**) reports a compositional map of the pollen grains, showing that chemical elements composing it mainly consist in sulfur (red), potassium (green), oxygen (blue) and phosphorus (light blue). Panels (**b**–**e**) report maps showing the homogeneous distribution of each single listed component. In order to compare the elemental composition of pristine pollen grains with Fe-decorated sporopollenins, the very same characterization was also performed on the latter. The measured compositional map is reported in panel (**f**), showing that along with the same elements composing pristine grains, also iron (purple) and chlorine (yellow) are found in the examined sporopollenins. Panels (**g**–**l**) report single maps showing each element composing the analyzed sample. In panels (**m**, **n**) compositional spectra are reported for a pristine grain sample and a Fe-decorated sporopollenin respectively. Remarkably, in the spectrum relative to the latter sample peaks relative to chlorine and iron are visible, while they are absent in the pristine grain spectrum.

Along with SEM morphological and dimension-wise analysis, compositional analysis via energy-dispersive X-ray spectroscopy (EDX) has been performed both on pristine pollen grains as well as Fe-decorated sporopollenins: in particular, four pristine grains and four functionalized samples have been analyzed, resulting in fully coherent measurement outcomes. The results are reported in Fig. 5, while more technical details about the technique and setup are reported in the “Experimental section”. As visible in the elemental composition maps reported in Fig. 5, panels from a to l, sulfur, potassium, oxygen and phosphorus are found in both pristine and functionalized samples. Concerning Fe-decorated sporopollenins, the presence of Fe and Cl is evident as shown in Fig. 5, panels f, k and l. Furthermore, no trace of iron nor chlorine is found in pristine pollen grains. In order to more quantitatively describe the elemental composition of the analyzed samples, compositional spectra are reported in panels m and n of Fig. 5. Here, is it possible to notice the absence of peaks relative of Fe and Cl in the pristine sample, and their presence in the compositional spectrum of Fe-decorated sporopollenins. It is worth noticing two features of the measured spectra: first, in the Fe-decorated sporopollenins spectra the peak relative to K at $\sim 3.314$ keV is absent, probably due to the reduced presence of K in the functionalized samples; second, in the measured spectra the peaks relative to carbon seem to be not resolved due to the presence of other elements having peaks with similar energies, but carbon is found in the quantitative analysis of the measured spectra, reported in Table 2.

**Table 2:** Quantitative analysis of acquired compositional spectrum measured for one of the analyzed samples of pristine pollen grains and Fe-decorated sporopollenin.

Element	Series	Pristine (wt%)	Pristine (norm. wt%)	Pristine (norm. at.%)	Fe-decorated (wt%)	Fe-decorated (norm. wt%)	Fe-decorated (norm. at.%)
Potassium	K-series	3.58	6.91	2.77	0.10	0.27	0.11
Sulfur	K-series	0.57	1.11	0.54	0.48	1.33	0.69
Oxygen	K-series	33.20	64.03	62.72	12.39	34.39	35.87
Phosphorus	K-series	1.629	3.14	1.59	1.96	5.44	2.93
Carbon	K-series	12.87	24.81	32.38	13.48	37.44	52.01
Chlorine	K-series	–	–	–	4.33	12.02	5.66
Iron	K-series	–	–	–	3.28	9.11	2.72
	Sum:	51.86	100.00	100.00	36.02	100.00	100.00

### Investigation of the magnetic properties of Fe-decorated SPs using superconducting quantum interference device (SQUID) magnetometry and electron paramagnetic resonance (EPR)

**Figure 6:**
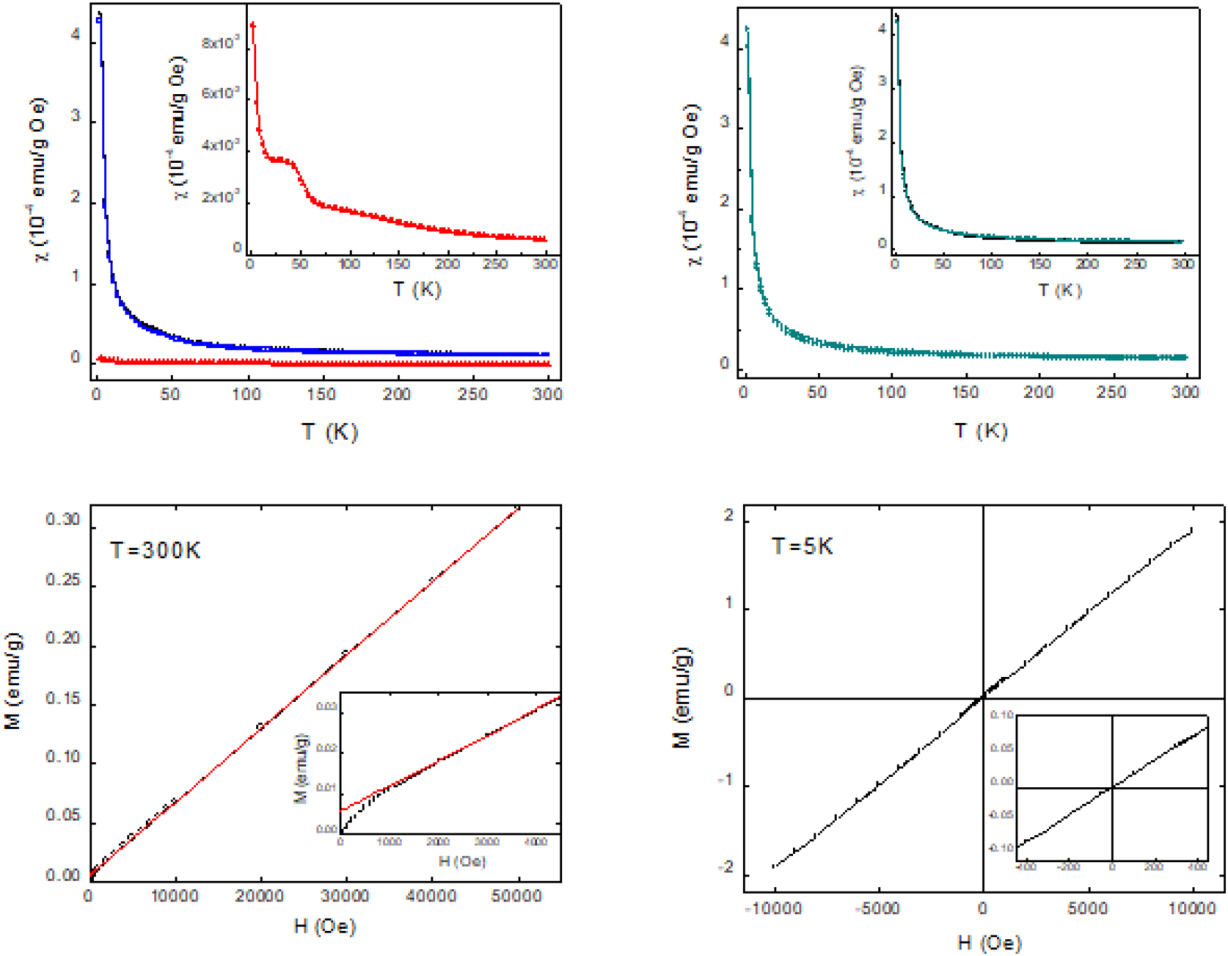
(Top-left) Temperature dependence of mass magnetic susceptibility at 1kOe for pristine (red curve) and IL-treated (black curve) samples. Blue curve represents the IL-treated curve after subtraction of pristine contribution. The Inset shows pristine χ-curve in an enlarged scale. (Top-right) ZFC and FC magnetic susceptibility for IL-treated sample at 20 Oe. In the Inset FC magnetic susceptibility at 20 Oe (green curve) and at 1 kOe (black curve) are compared. (Bottom) Magnetization as function of *H* measured at room temperature (bottom-left) and at 5 K (bottom-right) In the Insets an enlargement of the respective curve is reported in the low field region.

In Fig. 6, top-left panel, the field cooling (FC) magnetic susceptibility curves per unit mass, measured at 1 kOe, are shown for both samples: pristine (red curve) and IL-treated pollen grains (black curve). A markedly different behavior is evident at the lowest investigated temperatures, with χ-values two order of magnitude higher for the IL-treated pollen grain, indicating that iron atoms are actually incorporated in this sample. The curve obtained by subtracting the pristine contribution to IL-treated pollen grains one is also shown in the same figure (blue curve). In the inset, the χ curve for pristine is highlighted and reveals, at about 50 K, the typical paramagnetic contribution of the liquid phase of the residual oxygen present in the sample, superimposed to a weak paramagnetic trend. Radicals present in the SP, such as carotene and phenols, actually contain oxygen and could account for this contribution. Magnetic susceptibility of the IL-treated sample measured at *H* = 20 Oe in ZFC and FC regime is reported in Fig. 6, top-right panel. The ZFC and FC curves are practically indistinguishable and a comparison between the FC curves at 1kOe and at 20 Oe (inset of Fig. 6, top-right panel) shows that they very nicely overlap, too. These measurements highlight the typical Curie-like paramagnetic behavior of the sample. In Fig. 6, the bottom-left panel illustrates the magnetization measurements as a function of the magnetic field at *T* = 300 K for IL-treated sample. The fit of the linear part, accounting for a purely paramagnetic contribution, yields to a zero-field magnetization of about 6·10^–3^ emu/g, as evidenced in the Inset. This M(H = 0) value is consistent with the asymptotic χ-value in the limit of high temperatures. In principle, it may account for the magnetic contribution due to a magnetic phase that is saturated even at room temperature, which is superimposed to the dominant paramagnetic contribution of Fe in the sample. However, the amount of the phase giving rise to this saturated magnetic contribution can be considered almost negligible. For example, in the case of metallic Fe (M ≈ 222 emu/g, T_C_ ≈ 770 °C), its amount would be lower than 0.003% of the sample. Hysteresis cycle measured at 5 K and reported in Fig. 6, bottom-right panel, displays a clear linear trend consistent with the dominant paramagnetic contribution of iron in the sample. The linear trend is well evident also in the low field region, as shown in the Inset.

From the analysis of magnetic results, we can tentatively speculate a few possible microscopic mechanisms related to the Fe insertion in the IL-treated sample. The Curie constant value assessed from susceptibility curves allowed us to estimate the amount of the magnetic ions per unit volume of this sample, for which a density of ≈ 0.5 g/cm^3^ has been empirically evaluated. By assuming g = 2 and S = 2 or S = 5/2 (typical values for Fe^2+^ and Fe^3+^ ions in high spin (HS) configuration, respectively) we got approximately N = (1.1 ± 0.3) × 10^20^ Fe ions/cm^3^. Concerning this estimate, we should keep in mind that the N value increases if the density is larger than 0.5 g/cm^3^ and for lower spin values. A negligible value was obtained for the Weiss constant, confirming our sample is not far away from a pure paramagnetic system.

**Figure 7:**
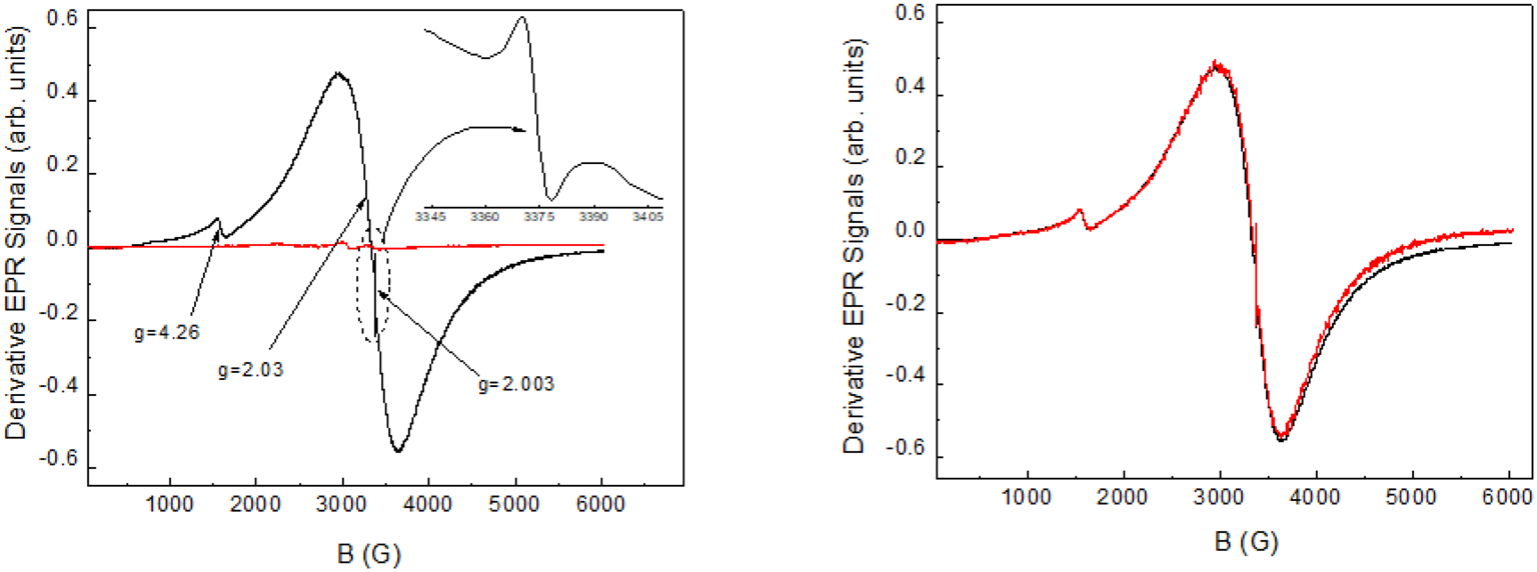
(Left) Derivative EPR signals measured in pristine (red line) and IL-treated (black line) samples. Signal centered at g = 2.003 is shown in enlarged scale. (Right) EPR spectra collected from two nominally identical IL-treated samples: Fe-SP1 (red line) and Fe-SP2 (black line).

The room temperature EPR spectra from pristine (red curve) and IL-treated (dark curve) samples, collected in the same experimental conditions and normalized to the sample mass, are directly compared in Fig. 7, left panel. The spectrum from IL-treated sample consists of two main contributions: a wide signal (∆*B* = 680 G) centered at g ≈ 2.03, attributable to Fe^3+^ paramagnetic ions in sample regions with a high Fe concentration, and a narrow signal (∆*B* ≈ 98 G) centered at g ≈ 4.26, typical of Fe^3+^ ions in structurally disordered systems, e.g. coming from Fe^3+^ ions in low symmetric, tetrahedrically or octahedrically coordinated, isolated sites in glass-like systems^30^. Besides, a very narrow signal (∆*B* = 8 G) at g = 2.003 is recorded, as shown in the figure’s enlargement. An EPR line at g = 2.003 is recorded from pristine sample, too, with very low intensity. We can tentatively ascribe this signal to traces of paramagnetic radicals present in the compound. Other resonances are recorded from pristine sample, with g values ranging between 2.2 and 2.8, with negligible intensities with respect to the features observed for the IL-treated sample. These signals are attributable to traces of unknown paramagnetic species.

It is worth mentioning that the IL-treated sample was prepared in two distinct batches, here labeled Fe-SP1 and Fe-SP2, in order to test the reproducibility of the magnetic properties induced by the functionalization of SP with ionic liquids. The EPR spectra measured for the two batches, normalized for the same experimental conditions and sample mass, overlap very well as highlighted in Fig. 7, right panel. A weak difference can be observed at high magnetic fields, which is due to an experimental drift more evident for sample Fe-SP1 due to its lower mass in the EPR cavity (mass of Fe-SP1 was ≈ 0.1 times the mass of sample Fe-SP2), which is corroborated by the trend of the signal-to-noise ratio. From the double integration of the derivative spectrum, the area of the absorption signal can be estimated and compared to the one obtained from an EPR standard (a Varian Pitch, in this case) in order to evaluate the amount of Fe^3+^ centers in the sample. Assuming the density of the IL-treated sample ≈ 0.5 g/cm^3^ and considering S = 5/2 (pertaining to the EPR active HS Fe^3+^ ion), the EPR signal corresponds to a number of paramagnetic centers (N) of (1.4 ± 0.2) × 10^20^/cm^3^. Again, the N value increases if the density is larger than 0.5 g/cm^3^ and for lower spin values. Finally, the order of magnitude of paramagnetic centers contributing to the narrowest signal centered at g = 2.003 for the IL treated sample can be evaluated from empirical calculations, obtaining ≈ 10^16^–10^17^ centers for 1 g of sample, depending on the spin value. Following the same empirical calculations, about 10^17^ Fe^3+^ ions (S = 5/2) can be estimated to be responsible for the signal centered at g ≈ 4.26. Concerning the estimation of Fe amount (N-value) in the IL-treated sample, a good agreement is found between the N values independently obtained by the two magnetic techniques. It has to be observed that, in principle, both Fe^2+^ and Fe^3+^ would contribute to the χ and *M* curves while, at the investigated temperature, the EPR technique is only sensitive to Fe^3+^. The good consistence between magnetization and EPR results then suggests that Fe entered the IL-treated sample mainly as Fe^3+^.

In conclusion, starting from pollen grains and using ionic liquids, we obtained Fe-decorated sporopollenins, preserving the integrity and morphology of the organic capsules, as demonstrated with scanning electron microscopy investigations. FT-IR suggested the presence of Fe in the capsule, which was confirmed by X-ray photoemission spectroscopy as well as EDX studies. The paramagnetic behavior of our Fe-functionalized sporopollenin was demonstrated thanks to electron paramagnetic resonance and magnetization measurements. The present strategy of combining ionic liquids’ chemical properties, which allow for sporopollenin isolation from pollen grain, together with the modification of magnetic properties is of interest in view of the simultaneous functionalization and field effect control of semiconductor-based nanodevices^31^. Besides, while nanoscale architectures based on inorganic systems functionalized with metals represent a solid playground for advanced studies across magnetism, electronics and photonics at large^32–35^, the Fe-functionalized organic microparticles investigated in the present work bear great potential for applications in biotechnology, biomedicine and microrobotic. Proof of concept and/or possible application of chemical modified pollen grains has been reported in literature and recently reviewed^36^. Our Fe-decorated microparticles, characterized by a remarkably narrow size dispersion, are expected to be compatible with human blood vessels for drug delivery of highly localized pharmaceutical agents in difficult-to-access regions^37^. On top of this, they can be used as magnetically-driven microtribology performing the cleaning and grinding of channels, spanning from blood vessels to pipelines. Finally, more possible applications can be envisioned in the context of magneto-hydrodynamic fractionation devices used for immunomagnetic affinity-based sorting of multiple biological substances^38^.

## Experimental section

### Materials and methods

Tree/shrub pollens from *Populus deltoides* (eastern cottonwood) were purchased from Sigma-Aldrich. All reagents and solvents employed were of analytical grade and were used without further purification. 1-Butyl-3-methylimidazolium tetrachloroferrate, [bmim][FeCl_4_], was prepared following a previously reported procedure^39^.

To 200 mg of *Populus deltoides* pollen grains, 2.0 g of [bmim][FeCl_4_] were added and the mixture was heated at 160 °C for 1.5 h. Sporopollenin microcapsule were collected by filtration using a glass filter funnel and filter crucible under vacuum, washed three times with distilled water (5 mL), and dried in an oven for 12 h at 50 °C and finally weighted. The resulting yield was 35%.

The ATR-FTIR spectra of the IL-treated pollen grains were obtained using a Cary 660 FTIR Spectrometer equipped with a micro-ATR slide accessory with a Germanium crystal. The spectra were measured in a 4,000–900 cm^−1^ range, with 32 scans for background and for samples.

XPS measurements were carried out in an ultra-high vacuum (UHV) chamber equipped with a non-monocromated X-ray source (VSW-TA10 Mg Kα radiation, 1,253.6 eV), operating at 120 W (12 kV and 10 mA) and a hemispherical analyser (VSW-HA100) with a 16-channel detector. The detector works at constant pass energy set to 22 eV. The collected spectra were fitted using CasaXPS software subtracting a Shirley’s type background^25^ and fitting the picks using mixed Gaussian and Lorentzian components. Spectra were calibrated imposing the 1*s* core transition of carbon at 284.8 eV^40^.

SEM images were acquired using a FEG-SEM Merlin from ZEISS. Typical imaging conditions were: magnification 1–3 × 10^4^, working distance 5–10 mm, ETH 5 kV, current 130 pA, secondary electron detector. The same imaging conditions have been reported elsewhere^13^.

We performed TEM–EDX investigation of our samples using a Zeiss Libra 120 microscope operating at an accelerating voltage of 120 keV, equipped with an in-column omega filter for energy-filtered imaging. EDX spectroscopy analyses (maps) were carried out on the same microscope working in scanning mode (STEM) with a HAADF detector thanks to a Bruker XFlash 6 T − 60 SDD detector.

Static magnetization measurements were performed by using a Quantum Design Squid magnetometer. Different magnetic fields were applied to study the temperature dependence of magnetization, M(T), in the range 2–300 K, both in zero field cooling (ZFC) and field cooling (FC) regimes. Magnetic field dependence of magnetization, M(H), was investigated at different temperatures with magnetic field ranging between 0 and ± 50,000 Oe. Quite similar parameters for magnetic characterization have been reported elsewhere^41^.

Electron paramagnetic resonance (EPR) measurements were carried out at about 9.4 GHz with a Bruker spectrometer. Particular care was devoted in determining the sample mass and position in the resonant cavity to compare signal intensities (areas) with that of a suitable standard (Varian Pitch) and to estimate the relative amount of the paramagnetic species in the samples.
