# Publisher Correction: Fe-functionalized paramagnetic sporopollenin from pollen grains: one-pot synthesis using ionic liquids

**DOI:** 10.1038/s41598-020-77728-1

**Published:** 2020-11-25

**Authors:** C. Chiappe, M. J. Rodriguez-Douton, M. C. Mozzati, D. Prete, A. Griesi, L. Guazzelli, M. Gemmi, S. Caporali, N. Calisi, C. S. Pomelli, F. Rossella

**Affiliations:** 1 Dipartimento Di Farmacia, Università Di Pisa, Via Bonanno 33, 56126 Pisa, Italy; 2 Dipartimento Di Fisica, Università Di Pavia, Via Bassi 6, 27100 Pavia, Italy; 3 NEST, Scuola Normale Superiore and Istituto Nanoscienze-CNR, Piazza San Silvestro 12, 56126 Pisa, Italy; 4 Center for Nanotechnology Innovation@NEST, Istituto Italiano Di Tecnologia, Piazza San Silvestro, 12, 56127 Pisa, Italy; 5 Department of Chemistry, Life Sciences and Environmental Sustainability, University of Parma, Parco Area delle Scienze 17/A, 43124 Parma, Italy; 6 Dipartimento Di Ingegneria Industriale, Università Di Firenze, Via di S. Marta 3, 50129 Firenze, Italy; 7 INSTM, Via Giusti 9, 50123 Firenze, Italy

Correction to:* Scientific Reports* 10.1038/s41598-020-68875-6, published online 20 July 2020

In the original version of this Article, F. Rossella was incorrectly listed as a corresponding author. The correct corresponding author for this Article is C.S. Pomelli. Correspondence and request for materials should be addressed to christian.pomelli@unipi.it.

This error has now been corrected in the HTML and PDF versions of the Article.

